# Stiff matrix-induced KRTAP2-3 expression suppresses ciliogenesis via actin tension-driven chromatin remodeling

**DOI:** 10.1038/s41419-026-08678-1

**Published:** 2026-04-02

**Authors:** Xiying Chen, Li Yi, Guangsong Xie, Hao Jin, Feng Yang, Wenjian Cao, Zhouyuanjing Shi, Zhangqi Xu, Shucan Li, Chunxiao Huo, Ya Li, Aifu Lin, Wei Liu, Guangshuo Ou, Tianhua Zhou, Baohua Ji, Shanshan Xie

**Affiliations:** 1 National Clinical Research Center for Children and Adolescents’ Health and Diseases, Children’s Hospital, Zhejiang University School of Medicine, Hangzhou, China; 2 Department of Cell Biology, Zhejiang University School of Medicine, Hangzhou, China; 3 Institute of Biomechanics and Applications, Department of Engineering Mechanics, Zhejiang University, Hangzhou, China; 4 Wenzhou Institute, University of Chinese Academy of Sciences, Wenzhou, China; 5 State Key Laboratory of Industrial Control Technology, Research Center for Analytical Instrumentation, Institute of Cyber-Systems and Control, Zhejiang University, Hangzhou, China; 6 Department of Gastroenterology, The Second Affiliated Hospital of Zhejiang University School of Medicine, Hangzhou, China; 7 MOE Laboratory of Biosystem Homeostasis and Protection, College of Life Sciences, Zhejiang University, Hangzhou, China; 8 Metabolic Medicine Center, International Institutes of Medicine and the Fourth Affiliated Hospital, Zhejiang University School of Medicine, Yiwu, China; 9 Tsinghua-Peking Center for Life Sciences, Ministry of Education Key Laboratory for Protein Science, School of Life Sciences, Tsinghua University, Beijing, China; 10 Center for RNA Medicine, International Institutes of Medicine, The Fourth Affiliated Hospital of Zhejiang University School of Medicine, Yiwu, China; 11 Eye Center, The Second Affiliated Hospital, Zhejiang University School of Medicine, Hangzhou, China

**Keywords:** Cell biology, Cytoskeleton

## Abstract

Primary cilia are sensory organelles that project from the cell surface and play vital roles in cell signaling pathways essential for development and homeostasis. However, the mechanotransduction pathways through which cells perceive and respond to matrix rigidity to regulate ciliogenesis remain poorly understood. In this study, we find that increased matrix stiffness significantly reduces primary cilia formation compared to soft matrix. Disruption of actin polarization of cells on stiff matrix restores ciliation, indicating the actin cytoskeleton as a pivotal transducer of mechanical signals in this process. RNA sequencing identifies significant upregulation of *KRTAP2-3* (keratin-associated protein 2-3) mRNA in cells on stiff matrix. Functional assays reveal that knockdown of *KRTAP2*-*3* reverses the stiffness-induced inhibition of ciliogenesis. Additionally, actin polarization on stiff matrix promotes *KRTAP2-3* expression, thereby inhibiting cilia formation. Further mechanistic studies show that actin cytoskeleton tension induces nuclear deformation and alters nuclear architecture, thereby enhancing chromatin accessibility at the *KRTAP2-3* gene locus, which leads to the activation of *KRTAP2-3* transcription. Collectively, these results suggest a previously unrecognized mechanotransduction pathway in which matrix stiffness drives actin cytoskeleton tension-dependent nuclear deformation, chromatin remodeling, and upregulation of *KRTAP2-3*, ultimately leading to the suppression of ciliogenesis.

## Introduction

Primary cilia are dynamic “antenna” organelles that sense changes in the extracellular environment and transmit signal information to regulate diverse cellular, developmental and physiological processes, by regulating multiple crucial signaling pathways [1]. Essential works have demonstrated the important roles of cilia in adipogenesis [2], meiosis [3], and circadian rhythms [4]. Consequently, mutations or perturbations that disrupt ciliary structure or function lead to a broad spectrum of disorders collectively known as ciliopathies, which affect multiple organs during embryonic development as well as postnatal life [3–5].

Beyond biochemical signals, cells are also highly sensitive to the physical properties of their microenvironment, among which the mechanical characteristics of the extracellular matrix (ECM) play a critical role. In particular, matrix stiffness has emerged as a key regulator of cellular behaviors, including proliferation, differentiation, morphogenesis, and cell fate determination [6, 7]. Recent studies have begun to extend the concept of mechanoregulation beyond transcriptional programs and cytoskeletal organization to include organelle structure and function, showing that matrix stiffness can influence mitochondrial morphology and activity as well as Golgi organization and positioning [8, 9]. However, the mechanisms by which cells sense and respond to matrix stiffness to regulate ciliogenesis remain poorly understood.

Cells sense and respond to the mechanical properties of their environment largely through the actin cytoskeleton [6, 7, 10–15]. Actin cytoskeleton created tension in response to external mechanical cues, which is proportional to its length according to the so-called the traction-distance law [16], leading to nuclear deformation and changes in nuclear architecture [17–20]. Such changes are known to impact gene expression by modulating chromatin accessibility and transcriptional activity [21–27]. However, the direct link between ECM stiffness, actin polarization, and ciliogenesis remains unclear. Specifically, how actin-mediated nuclear deformation and chromatin remodeling regulate the expression of genes involved in ciliogenesis in response to matrix stiffness has not been fully elucidated.

In this study, we show that stiff matrix inhibits ciliogenesis, but disrupting actin polymerization restores ciliation, highlighting actin’s role as a crucial mechanosensory and regulator. RNA sequencing reveals upregulation of *KRTAP2-3* on stiff matrix, and its knockdown rescues cilia loss. Actin polarization on stiff matrix enhances *KRTAP2-3* expression by inducing nuclear deformation and increasing chromatin accessibility at its locus. These findings define a mechanotransduction pathway in which matrix stiffness drives actin cytoskeleton tension-mediated nuclear deformation and chromatin remodeling to upregulate *KRTAP2-3*, thereby suppressing ciliogenesis.

## Materials and methods

### Cell culture

For all experiments assessing ciliogenesis, hTERT RPE-1 cells (CRL-4000, ATCC) were cultured in DMEM/F12 medium (C11330500BT, Gibco) with 10% fetal bovine serum (FSP500, ExCell Bio) and 0.01 mg/mL hygromycin (S2908, Selleck). 3T3 Swiss albino cells (gifted by Dr. Wei Rao, Zhejiang University) were cultured in DMEM high glucose medium (C11995500BT, Gibco) with 10% fetal bovine serum. All cells were maintained at 37 °C in a humidified incubator with 5% CO_2_.

For experiments performed on polyacrylamide hydrogels, cells were seeded at the following densities: RPE-1 cells, ~2.5 × 10⁵ cells/mL; 3T3 Swiss cells, ~3.0 × 10⁵ cells/mL. For experiments performed on cell culture plate, RPE-1 cells were seeded at ~2.5 × 10⁵ cells/mL. For *KRTAP2-3* knockdown transfection experiments, transfections were carried out when cells confluence reached approximately 70%.

### Transfection

For transient knockdown, siRNAs targeting human *KRTAP2-3* (synthesized by GenePharma) were transfected into cells with Lipofectamine RNAiMAX (13778150, Invitrogen) for 48 h according to the manufacturer’s instructions. The siRNA sequences were as follows: 5′-CUGCCAGCCUGUGUCUGUGTT-3′ (si*KRTAP2-3* 1), 5′-CCAGGAUAAUACUAUUUGUTT-3′ (si*KRTAP2-3* 2).

### Drug treatment

For drug treatments, cells were treated with cytochalasin B (1 μM), cytochalasin D (0.02 μM), latrunculin A (0.05 μM, 0.1 μM), or blebbistatin (5 μg/mL, 10 μg/mL) for 6 h in well plates. For treatments in gels, cells were treated with cytochalasin B (1 μM), cytochalasin D (0.02 μM), latrunculin A (0.2 μM), or blebbistatin (10 μg/mL) for 6 h and allowed a 6 h recovery after washout. For *KRTAP2-3* overexpression combined with drug treatments, *KRTAP2-3* was cloned into the pVAX-V5 vector (gifted by Dr. Na Kong, Zhejiang University), transcribed using a T7 High Yield RNA Transcription Kit (TR101, Vazyme), and stored at −80 °C. mRNA transfection was performed with Lipofectamine MessengerMAX (LMRNA015, Invitrogen) for 12 h, concurrent with cytochalasin B treatment.

### Immunofluorescence staining and image analysis

Cells were seeded on collagen-coated (C3867-1VL, Sigma) polyacrylamide (PA) gels or coverslips (10210012CE, CITOTEST) at specified densities, followed by transfection and/or treatments as described before. Cells were fixed with cold methanol for 10 min at −20 °C or with 4% paraformaldehyde for 10 min at room temperature. Fixed cells were then permeabilized with 0.2% Triton X-100 in PBS for 15 min and blocked with 5% BSA in 0.2% PBST for 1 h.

Primary antibodies, diluted in blocking solution, were applied for 2–3 h at room temperature, followed by incubation with secondary antibodies for 1 h. Primary antibodies used included anti-ARL13B (17711-1-AP/CL488-17711/CL647-17711, Proteintech Group), anti-γ-tubulin (T6557, Sigma) and anti-V5 tag (R960-25, Invitrogen). Secondary antibodies used were Alexa Fluor 488 donkey anti-rabbit IgG (A32790, Invitrogen), Alexa Fluor 555 donkey anti-mouse IgG (A32773, Invitrogen), and Alexa Fluor 647 goat anti-mouse IgG (A32787, Invitrogen). Actin filaments were stained with ActinRed 555 (R37112, Invitrogen) or TRITC Phalloidin (40734ES75, Yeasen). Nuclei were stained with DAPI (C1002, Beyotime).

Images were captured using an OLYMPUS FV3000 laser scanning confocal microscope and an OLYMPUS Spin10 spinning disk confocal microscope with a 60× oil objective for coverslips. For PA gels, images were captured using an OLYMPUS BX61 upright laser scanning confocal microscope with a 60× water objective. Quantitative analysis of actin filaments length and number were performed on images of phalloidin-stained cells using the FSegment MATLAB script developed by Rogge et al. Quantitative analysis of actin filaments alignment was performed on images of phalloidin-stained cells using the OrientationJ software. The displayed representative images were adjusted for contrast.

### RNA extraction and quantitative real-time PCR

Total RNA was extracted using TRIzol reagent (15596018CN, Invitrogen) according to the manufacturer’s instructions, with DNase I treatment (AM2239, Invitrogen) to prevent DNA contamination. RNA was then reverse transcribed into cDNA using the HiScript II Q RT SuperMix (R222-01, Vazyme). Quantitative RT-PCR (qRT-PCR) was performed on a CFX-Touch System (Bio-Rad) or LightCycler® 480 Instrument II (Roche) using ChamQ Universal SYBR qPCR Master Mix (Q711-02, Vazyme). Each reaction was run in triplicate, with *GAPDH* serving as the internal control. Primer sequences used for this study are provided in Table S2.

### Polyacrylamide gel preparation

Polyacrylamide gels were prepared following a modified version of a previously described protocol [28, 29]. Briefly, coverslips were treated with 0.1 N NaOH and air-dried. A thin layer of 3-aminopropyltrimethoxysilane (281778, Sigma) was applied evenly and allowed to sit for 6 min, followed by a distilled H₂O rinse. Coverslips were then immersed for 30 min in 0.5% glutaraldehyde (G6257, Sigma) in PBS, rinsed with distilled H₂O, and air-dried.

The acrylamide (A4058, Sigma) and bis-acrylamide (M1533, Sigma) mixture was prepared according to Fig. S1, with 0.5% ammonium persulfate (A500857, Sangon) and 0.05% TEMED (ST728, Beyotime) as initiators. The mixture was applied to larger coverslips and immediately covered with the prepared coverslips. After polymerization, the larger coverslips were removed, and the gels were rinsed in 100 mM HEPES (V900477, Sigma).

The polymerized gels were activated under UV light for 6 min using Sulfo-SANPAH (22589, Thermo Scientific) in 100 mM Hepes, with activation repeated once more. Gels were then incubated overnight in 0.2 mg/mL collagen solution, followed by removal of excess collagen and PBS washing. Prepared gels were stored in PBS at 4 °C and sterilized by UV before cell seeding.

### Measurement of Young’s modulus

Young’s modulus of polyacrylamide gels was measured using the nanoindenter (PIUMA). By moving the probe at a constant speed, a controllable force was applied to the sample in the z-direction. This would cause an indentation on the sample, and the depth of the indentation could be recorded as a function of the force, by which the depth-force curve was obtained. The relationship between force and indentation depth could be represented by the Hertz model, the Young’s modulus of the sample could be obtained by using the least mean square fit of the Hertz model. These measurement data were provided by shinning tech company (China).

### RNA extraction, library preparation, and sequencing

RPE-1 cells were seeded on polyacrylamide gels for 12 h. The gels, along with attached cells, were removed from the coverslips using a cell scraper and immediately immersed in TRIzol reagent. Total RNA was extracted from cells grown on gels with varying elastic moduli. RNA samples were then prepared for library construction and sequencing by LianChuan Bio (China).

### RNA-seq differentially expressed genes analysis

The raw sequencing data were trimmed using Cutadapt (version 1.9). Clean reads were then aligned to the GRCh38 genome using HISAT2 (version 2.2.1). Transcript assembly was performed with StringTie (version 2.1.6) using default parameters. Uniquely mapped reads were utilized to calculate gene counts and FPKM (fragments per kilobase of transcript per million mapped reads). Differential expression analysis between the two groups was conducted using DESeq2. The selection criteria for differentially expressed genes (DEGs) in this study were an adjusted *p*-value < 0.05 and an absolute Log_2_ fold change >1. Two independent biological replicates were generated and analyzed for each stiffness condition in the RNA-seq experiments.

### Clustering analysis of gene expression across stiffness gradients

**Fig. 1:**
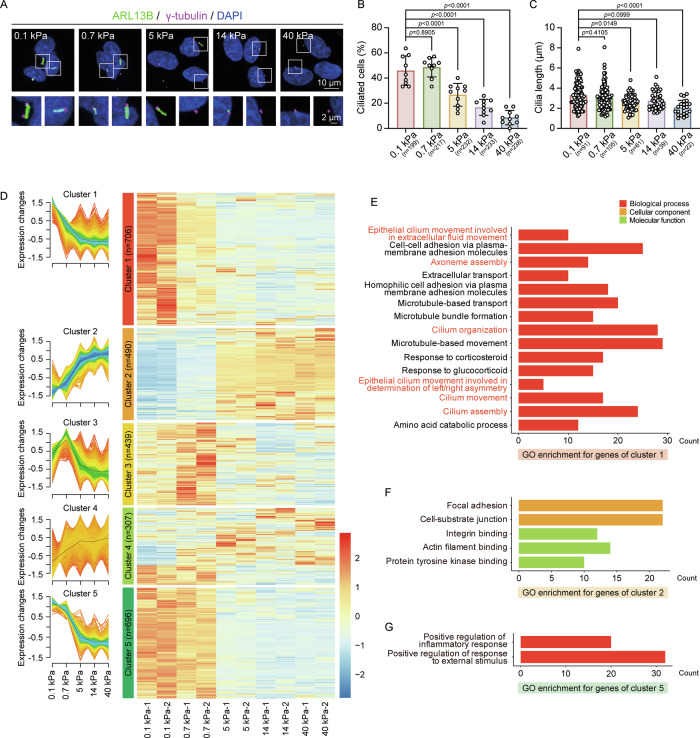
Matrix stiffness inhibits ciliogenesis in mammalian cells. RPE-1 cells were cultured on matrix of the indicated stiffness for 12 h and immunostained to analyze cilia formation. **A** Representative images show cells stained with anti-ARL13B (green) for cilia and anti-γ-tubulin (magenta) for basal bodies. Scale bars, 10 μm (upper panels) and 2 μm (lower panels). **B** Quantification of the percentage of ciliated cells under varied matrix stiffness (*n* = cell number). Quantitative data are presented as mean ± SD, ordinary ANOVA analysis. **C** Measurement of cilia length (*n* = cilia number). Quantitative data are presented as mean ± SD, Kruskal–Wallis test. Experiments were performed with at least three biological replicates. **D** Gene expression patterns analyzed by Mfuzz clustering, revealing five distinct expression clusters based on dynamic response to matrix stiffness. The left panel shows expression changes of rhythmic genes in each cluster, while the right panel displays a heatmap of *z*-score normalized expression (*p* < 0.05, |Log₂ fold change| > 1). Gene Ontology (GO) enrichment analysis of differentially expressed genes in clusters 1 (**E**), 2 (**F**), and 5 (**G**). GO terms meeting the enrichment threshold (*p* < 0.01) indicate processes regulated by matrix stiffness.

Differentially expressed genes from pairwise comparisons of the five stiffness-treated cells (0.1, 0.7, 5, 14, and 40 kPa) were subjected to clustering analysis. Clustering was performed on normalized counts using the fuzzy C-means algorithm in the Mfuzz R package (version 2.62). DEGs were assigned to 5 clusters, with the fuzzifier coefficient (*m*) set to 2.027. Cluster profiles are shown in Fig. 1D, and the gene membership lists for each cluster are provided in Table S1. Gene Ontology (GO) enrichment analysis for each cluster was conducted using the clusterProfiler R package (version 4.10.1).

### Differential gene expression on 40 kPa and 0.1 kPa matrix

Analysis of DEGs between cells on 40 kPa and 0.1 kPa matrix in our RNA-seq data was performed by using heatmap tools in Hiplot Pro (https://hiplot.com.cn/), a comprehensive web service for biomedical data analysis and visualization.

### Calculation of nuclear deformation and stress

We constructed a FEM model for calculating the nuclear deformation and stress. In the model, the cell is divided into four parts: cell membrane, cytoplasm, cell nucleus, and actin filaments (Fig. 5A). The cell membrane was modeled as an elastic shell with a thickness of 0.2 μm, Young’s modulus of 7 kPa, and Poisson’s ratio of 0.3 [30]. The cytoplasm and nucleus are modeled as elastomer. We took the Young’s modulus and Poisson’s ratio respectively as 0.25 kPa and 0.3 for the cytoplasm and as 1 kPa and 0.45 for the nucleus [31]. The geometric parameters in the model are derived from experimental observations. We assumed that the shape of the cell body is a semi-ellipsoid controlled by the spread area (Fig. S6A) and aspect ratio (Fig. S6B) and that of the cell nucleus is spherical before deformation. For the nucleus of a volume of 660 μm^3^, the radius is estimated as 5.5 μm.

All actin filaments in the cells were modeled as trusses without shear stress and with 20% pre-strain [32, 33] so that there is tension in the filaments. The Young’s modulus, Poisson’s ratio, and cross-sectional radius of the filaments are 250 kPa, 0.3, 0.25 μm [32], respectively. The actin filaments in the cell were simplified into three types: basal actin filaments connecting the cell nucleus and the cell membrane [34], basal actin filaments crossing above the cell nucleus, and the ventral actin filaments (Fig. S6C) [35]. For the ventral filaments under the cell nucleus, we modeled them as a fiber network. The distribution of the basal actin filaments including those connecting the cell nucleus and cell membrane and those crossing above the cell nucleus is generated according to the basal actin filament numbers (Fig. S6D) and alignment parameters (Fig. S6E) measured by the experiment. The result of each randomly generated distribution corresponds to a statistical data point.

The stiffness of the substrate was considered by a two-spring model [36], in which the focal adhesion and the substrate equivalent spring are connected in series to the fiber end. And we can denote the effective stiffness of the two-spring model as ${{\bf{k}}}_{{\bf{eff}}}={\left(\frac{{\bf{1}}}{{{\bf{k}}}_{{\bf{a}}}}+\frac{{\bf{1}}-{{\bf{v}}}_{{\bf{s}}}^{{\bf{2}}}}{{{\bf{E}}}_{{\bf{s}}}{\bf{d}}}\right)}^{-{\bf{1}}}$, where ${{\bf{k}}}_{{\bf{a}}}$ is spring stiffness of focal adhesions, ${{\bf{E}}}_{{\bf{s}}}$ and ${{\bf{v}}}_{{\bf{s}}}$ are Young’s modulus and Poisson’s ratio of substrate, respectively, **d** is the diameter of the focal adhesions [37]. The ends of the fibers are connected to a double spring system on substrates of different stiffnesses. The force is applied to the cell nucleus in the form of fiber pre-strain to cause the nucleus to deform. The stress and strain of the nucleus can be calculated by software. All these parameters were summarized in Table S3.

### Transposome assembly

Assembly of the Tn5 transposome with preannealed Mosaic End double-stranded (MEDS) oligonucleotides was carried out as previously described [38]. Briefly, equimolar amounts of Tn5 and a mixture of preannealed Tn5MEDS-A and Tn5MEDS-B oligonucleotides were combined and incubated for 60 min at room temperature. The assembled transposome was stored at −20 °C. The oligonucleotide sequences used were as follows: Tn5ME-A: TCGTCGGCAGCGTCAGATGTGTATAAGAGACAG; Tn5ME-B: GTCTCGTGGGCTCGGAGATGTGTATAAGAGACAG; pME_rev_block: /5’PHO/CTGTCTCTTATACA/3’ddC/.

### Omni-ATAC-seq

Omni-ATAC-seq was performed following a previously established protocol [39]. RPE-1 cells were seeded on the 0.1 kPa gels or 40 kPa gels for 12 h. Cells were digested and resuspended in cold PBS. 50,000 viable cells were centrifuged at 500 RCF at 4 °C for 5 min. After aspirating all supernatant, cell pellets were resuspended in 50 μL of cold ATAC-Resuspension Buffer (RSB; 10 mM Tris-HCl pH 7.4, 10 mM NaCl, 3 mM MgCl_2_ and double distilled water) containing 0.1% NP-40 (A600385, Sangon), 0.1% Tween-20 (A600560, Sangon), and 0.01% digitonin (D141, Sigma) and incubated on ice for 3 min. 1 mL of cold ATAC-RSB containing 0.1% Tween-20 was added, and then nuclei were centrifuged at 500 RCF for 10 min at 4 °C. Nuclei were resuspended in 50 μL of transposition mix containing 2× TD buffer (20 mM Tris-HCl pH 7.6, 10 mM MgCl_2_, 20% Dimethyl Formamide, and double distilled water), transposome, 33% PBS, 0.01% digitonin, 0.1% Tween-20 and double distilled water. Transposition reactions were incubated at 37 °C for 30 min with shaking. Reactions were cleaned up with AxyPrep PCR Cleanup Kit (AP-PCR-250, Axygen). The elution products were amplified with NEBNext Ultra II Q5 Master Mix (M0544S, NEB) and Nextera PCR primers. The Omni-ATAC-seq library was purified and subjected to high throughput sequencing on Illumina Novaseq 6000 platform.

### ATAC-seq data analysis

Adaptor sequences from ATAC-seq reads were trimmed using Cutadapt, and the reads were aligned to the hg19 reference genome using Bowtie2 [40]. Reads mapped to mitochondria, PCR duplicates, multi-mapped reads and reads mapped to ENCODE blacklist regions were removed. The quality control of ATAC-seq data was performed using the ATACseqQC package [41]. Reads from technical replicates of each condition were combined for peak calling by MACS2 [42] with narrow peak mode. Peaks from two conditions were merged to generate a union peak set. All peaks were annotated by HOMER [43]. FeatureCounts [44] was used to generate ATAC count matrix. edgeR package [45] was used for normalization and differential peak analysis. The read density profiles of the differential peaks were plotted by deepTools [46].

### Statistical analysis

Statistical analyses were performed using GraphPad Prism (version 8.4.0, GraphPad Software) using Student’s *t* test with a two-tailed approach and one-way ANOVA analysis. When data followed a normal distribution and met the assumption of homogeneity of variance, ordinary ANOVA was used; if the data were normally distributed but violated the assumption of homogeneity of variance, the Brown–Forsythe ANOVA test was applied; if the data did not conform to a normal distribution, the Kruskal–Wallis test was performed, followed by multiple comparisons where all matrix stiffness groups were compared with the 0.1 kPa group. For correlation analysis, a simple linear regression was used to derive the linear regression equation, and correlation analysis was performed to obtain the *p*-value and Pearson correlation coefficient *r*. Data are presented as means ± SD, and *p*-value is shown in the figures directly. Biological replicates are presented in the figure legends. Data points represent values from each cell, cilium, nucleus or experiment as detailed in the figure legends.

## Results

### Stiff matrix suppresses primary cilia formation

To explore how matrix stiffness regulates ciliogenesis in mammalian cells, we utilized a collagen-coated polyacrylamide gel system with controlled stiffness levels, calibrated to elastic moduli of 0.1, 0.7, 5, 14, and 40 kPa [28, 29]. These moduli mimic the physiological rigidity of native tissues, such as the brain (0.1–1.0 kPa), lung (0.44–7.5 kPa), muscle (8.0–17.0 kPa), and bone (*E* ~ 40 kPa) [47, 48]. We initially measured Young’s modulus of these gels using a nanoindenter (Fig. S1). hTERT RPE-1 cells were then cultured on these varying stiffness gels. Immunostaining revealed a significant, gradual decrease in the percentage of ciliated cells with increasing stiffness, while cilia length remained relatively constant (Fig. 1A–C). Similarly, mouse embryonic fibroblast 3T3-Swiss albino cells cultured on stiff matrix exhibited decreased ciliation with no significant changes in cilia length (Fig. S2A–S2C).

To investigate the underlying biological processes affected by matrix stiffness, we conducted RNA-seq analysis. Differentially expressed genes (|Log_2_ fold change| > 1, *p*-value < 0.05) from all pairwise comparisons were grouped into five clusters through Mfuzz clustering (Fig. 1D and Table S1). Clusters 1 and 5 exhibited a gradual decrease in gene expression with increasing stiffness, whereas Cluster 2 showed a gradual increase (Fig. 1D). Gene Ontology enrichment analysis revealed that genes in Cluster 1, enriched in “axoneme assembly”, “cilium organization”, and “cilium assembly” (Fig. 1E), align with the observed suppression of ciliation in stiff matrix. Additionally, Cluster 2 showed increased expression of genes associated with the actin cytoskeleton and focal adhesion complexes (Fig. 1F). These results suggest that matrix stiffness inhibits ciliation, likely through mechanotransduction pathways involving the actin cytoskeleton and cellular adhesions.

### Stiff matrix inhibits ciliogenesis by promoting actin polymerization

**Fig. 2:**
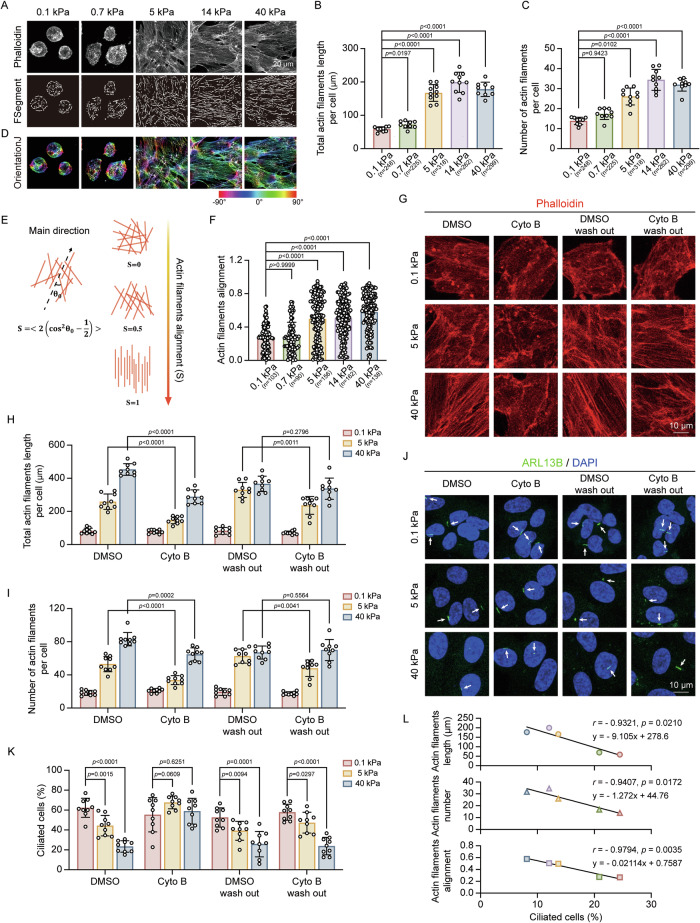
Actin cytoskeleton remodeling in response to different matrix stiffness. **A**–**F** RPE-1 cells were cultured on matrix of the indicated stiffness for 12 h and stained with phalloidin (grays) to visualize actin filaments. **A** Representative images showing actin polarization. FSegment line represents the trace of actin filaments automatically analyzed by FSegment algorithm. Scale bars, 20 μm. **B** Quantification of actin filaments length per cell (μm) (*n* = cell number), and quantitative data are presented as mean ± SD, Brown-Forsythe ANOVA analysis. **C** Quantification of actin filaments number per cell (*n* = cell number). Quantitative data are presented as mean ± SD, Kruskal–Wallis test. **D** OrientationJ line displays a visual representation of the orientation of actin filaments performed by OrientationJ method. **E** Quantification of actin filaments alignment using OrientationJ method defined as the polarization parameter (*S*), with *S* = 0 indicating disordered alignment and *S* = 1 indicating perfect alignment. **F** Quantification of actin filaments polarization (*n* = cell number). Quantitative data are presented as mean ± SD, Kruskal–Wallis test. Experiments were performed with at least three biological replicates. **G**–**K** RPE-1 cells grown on matrix of the indicated stiffness were treated with DMSO or cytochalasin B (Cyto B) for 6 h, followed by a 6 h recovery, and stained for actin and cilia. **G** Representative actin images following treatment. Scale bars, 10 μm. **H** Quantification of actin filaments length per cell (μm). **I** Quantification of actin filaments number per cell. The cell counts of (**H**) and (**I**) from left to right are as follows: DMSO: 343, 240, 204; Cyto B: 309, 331, 276; DMSO wash out: 267, 295, 307; Cyto B wash out: 302, 320, 266. **J** Representative images of cilia stained with anti-ARL13B (green). Scale bars, 10 μm. **K** Quantification of the percentage of ciliated cells post-treatment. The cell counts of (**K**) from left to right are as follows: DMSO: 343, 240, 204; Cyto B: 309, 331, 276; DMSO wash out: 267, 295, 307; Cyto B wash out: 302, 320, 266. Quantitative data are presented as mean ± SD, two-tailed Student’s *t* test. Experiments were performed with at least three biological replicates. **L** Correlation analysis between actin filaments characteristics (length, number, and alignment) and percentage of ciliated cells. Data are presented as simple linear regression and correlation analysis.

To elucidate the mechanisms by which matrix stiffness affects ciliogenesis, we investigated the role of actin polarization, which includes actin polymerization and alignment, as Cluster 2 genes were closely associated with the actin cytoskeleton and focal adhesions (Fig. 1F). Given that actin polymerization inhibits cilia formation while actin depolymerization promotes ciliogenesis [49], we hypothesized that changes in actin polarization may mediate the ciliary phenotype observed on stiff matrix. To explore this, we profiled actin polarization by analyzing the length, number, and alignment of actin filaments. An automated actin analysis algorithm named FSegment [50] was used to quantify actin filaments length and number (Fig. 2A). Results showed that cells display longer and more actin filaments on stiff matrix (Fig. 2A–C). Additionally, the alignment parameter (S), calculated by OrientationJ software [51–53], derived from the gradient structure tensor, was used to assess filaments alignment (Fig. 2D, E). On stiff matrix, actin filaments were found to be more aligned compared to those on soft matrix (Fig. 2F). These data suggest that stiff matrix promotes actin polarization.

To assess whether disrupting actin polarization would restore ciliation, cells were treated with cytochalasin B, an inhibitor of actin polymerization that blocks the addition of actin monomers to filament barbed ends [54]. This treatment reduced filaments length and number (Fig. 2G–I). Moreover, cytochalasin B treatment attenuated the differences in ciliation percentages across matrix with stiffness levels of stiff (40 kPa), intermediate (5 kPa), and soft (0.1 kPa) matrix (Fig. 2J, K). Upon cytochalasin B washout, stiffness-dependent variations in ciliation re-emerged (Fig. 2K). Similarly, cytochalasin D, which disrupts the interaction of G-actin and F-actin with cofilin [55], partially reduced stiffness-dependent differences in ciliogenesis (Fig. S3A, B). Additionally, correlation analysis confirmed significant associations between actin polarization and the percentage of ciliated cells (Fig. 2L). These data suggest that the actin cytoskeleton acts as a mechanosensor and actuator, mediating the impact of matrix stiffness on ciliary growth.

### Upregulated *KRTAP2-3* mRNA levels suppress primary cilia formation on stiff matrix

**Fig. 3:**
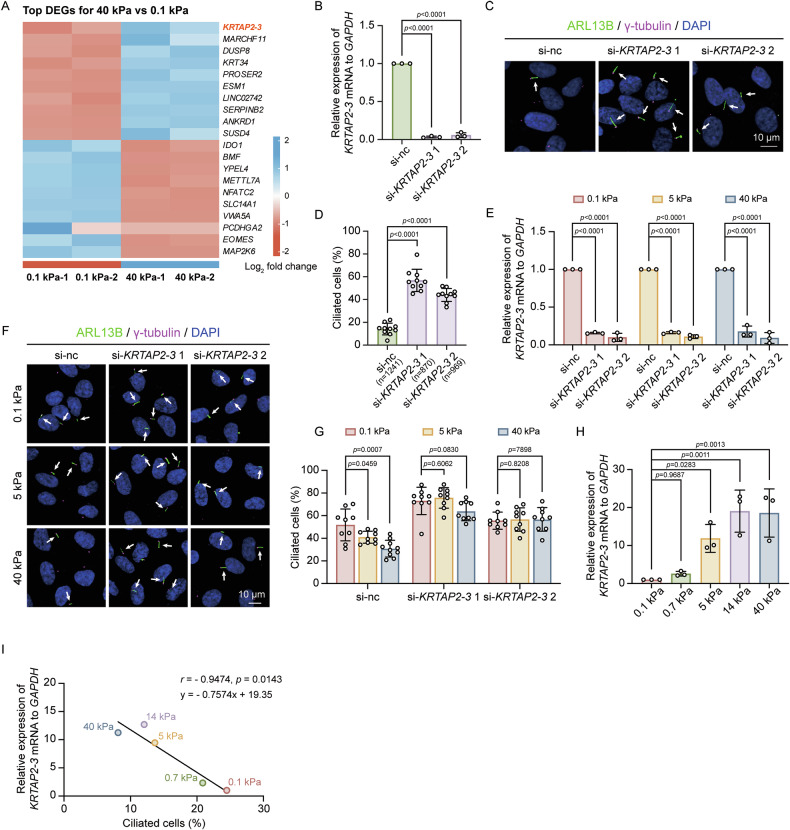
Role of *KRTAP2-3* in stiffness-induced suppression of ciliogenesis. **A** Heatmap of differentially expressed genes in cells cultured on 0.1 vs. 40 kPa matrix, with a threshold of |Log₂ fold change| > 1, *p* < 0.01, and gene counts >10. **B**–**D** RPE-1 cells transfected with control or *KRTAP2-3* siRNA for 48 hours were analyzed by qRT-PCR and immunofluorescence. **B** qRT-PCR of *KRTAP2-3* expression normalized to *GAPDH*. **C** Representative images showing cilia stained with anti-ARL13B (green) and anti-γ-tubulin (magenta). Scale bars, 10 μm. **D** Quantification of the percentage of ciliated cells (*n* = cell number). Quantitative data are presented as mean ± SD, two-tailed Student’s *t* test. Experiments were performed with at least three biological replicates. **E**–**G** RPE-1 cells transfected with siRNAs for 36 h were then grown on matrix of 0.1, 5, and 40 kPa stiffness for 12 h, followed by qRT-PCR (**E**) and immunofluorescence (**F**, **G**) to assess *KRTAP2-3* expression and ciliation. The cell counts of (**G**) from left to right are as follows: si-nc: 358, 382, 358; si-*KRTAP2-3* 1: 311, 329, 351; si-*KRTAP2-3* 2: 339, 344, 385. Quantitative data are presented as mean ± SD, two-tailed Student’s *t* test. Experiments were performed with at least three biological replicates. **H** *KRTAP2-3* mRNA expression in cells cultured on matrix of the indicated stiffness, quantified by qRT-PCR (*GAPDH* as control). Quantitative data are presented as mean ± SD, ordinary ANOVA analysis. Experiments were performed with at least three biological replicates. **I** Correlation analysis between *KRTAP2-3* mRNA expression and percentage of ciliated cells. Data are presented as simple linear regression and correlation analysis.

To uncover the downstream pathways through which actin mechanosensory signaling affects ciliogenesis, we performed differential gene expression analysis between cells on stiff and soft matrix by using our RNA-seq data. We identified *KRTAP2-3* as a significantly upregulated gene in cells grown on stiff matrix (Figs. 3A and S4A). To explore its role in stiffness-mediated ciliogenesis, we knocked down *KRTAP2-3* and observed a significant increase in the proportion of ciliated cells (Fig. 3B–D). Importantly, *KRTAP2-3* knockdown rescued the ciliation induced by stiff and intermediate matrix relative to soft matrix (Fig. 3E–G). In addition, overexpression of *KRTAP2-3* significantly inhibited primary cilia formation even on soft matrix (Fig. S4B, C), demonstrating that elevated *KRTAP2-3* levels are sufficient to suppress ciliogenesis in a mechanically permissive environment. Consistent with these functional results, *KRTAP2-3* mRNA levels were progressively increased with matrix stiffness (Fig. 3H), inversely correlating with ciliation levels (Fig. 3I). These results reveal the critical role of *KRTAP2-3* in mediating the inhibitory effects of matrix stiffness on cilia formation.

### Actin polymerization mediates the upregulation of *KRTAP2-3* mRNA induced by stiff matrix

**Fig. 4:**
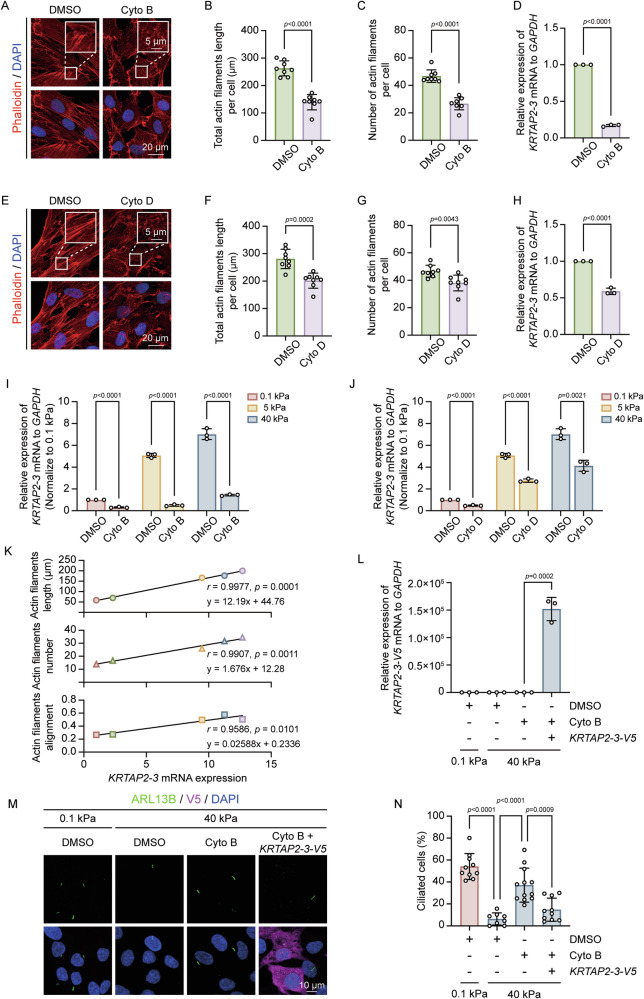
Actin polymerization regulates *KRTAP2-3* expression in response to matrix stiffness. **A**–**D** RPE-1 cells treated with DMSO or cytochalasin B (Cyto B) for 6 h were analyzed by immunofluorescence and qRT-PCR. **A** Representative actin images after treatment, scale bars, 5 μm (upper panels) and 20 μm (lower panels), **B** quantification of actin filaments length per cell (μm), **C** quantification of actin filaments number per cell, and **D** qRT-PCR of *KRTAP2-3* expression normalized to *GAPDH*. The cell counts of (**B**, **C**) from left to right are as follows: 257, 338. Quantitative data are presented as mean ± SD, two-tailed Student’s *t* test. Experiments were performed with at least three biological replicates. **E**–**H** RPE-1 cells treated similarly with cytochalasin D (Cyto D) were analyzed for actin polymerization and *KRTAP2-3* expression. **E** Representative actin images after treatment, scale bars, 5 μm (upper panels) and 20 μm (lower panels) **F** quantification of actin filaments length per cell (μm), **G** quantification of actin filaments number per cell, and **H** qRT-PCR of *KRTAP2-3* expression normalized to *GAPDH*. The cell counts of (**F**, **G**) from left to right are as follows: 282 and 286. Quantitative data are presented as mean ± SD, two-tailed Student’s *t* test. Experiments were performed with at least three biological replicates. **I**, **J** RPE-1 cells cultured on matrix of 0.1, 5, and 40 kPa stiffness were treated with DMSO, Cyto B, or Cyto D for 6 h, followed by qRT-PCR of *KRTAP2-3* expression (*GAPDH* as control). Quantitative data are presented as mean ± SD, two-tailed Student’s *t* test. Experiments were performed with at least three biological replicates. **K** Correlation analysis between actin filaments characteristics (length, number, and alignment) and *KRTAP2-3* expression levels. Data are presented as simple linear regression and correlation analysis. **L**–**N** RPE-1 cells transfected with *KRTAP2-3-V5* mRNA were treated with DMSO or Cyto B for 12 h on matrix of the indicated stiffness. **L** qRT-PCR analysis of exogenous *KRTAP2-3-V5*, normalized to *GAPDH*. **M** Representative images show cells with the indicated treatment stained with anti-ARL13B (green) for cilia and anti-V5 (magenta) for KRTAP2-3-V5. Scale bars, 10 μm. **N** Quantification of the percentage of ciliated cells. The cell counts of (**N**) from left to right are as follows: 449, 341, 531, 194. Quantitative data are presented as mean ± SD, two-tailed Student’s *t* test. Experiments were performed with at least three biological replicates.

Given that changes in both actin polarization and *KRTAP2-3* transcription influence the ciliary phenotype across different matrix stiffness levels, we investigated whether actin polarization could regulate *KRTAP2-3* mRNA expression. We used cytochalasin B and cytochalasin D to disrupt actin polymerization (Fig. 4A–C, E–G). These treatments significantly reduced *KRTAP2-3* mRNA levels (Fig. 4D, H). Notably, the difference in *KRTAP2-3* mRNA levels between 0.1 kPa, 0.5 kPa, and 40 kPa matrix was attenuated by inhibition of actin polymerization (Fig. 4I, J). Similarly, inhibition of actin polymerization by either latrunculin A (which sequesters actin monomers and hinders polymerization) or blebbistatin (which inhibits myosin II activity and thus actomyosin contractility) also narrowed the discrepancy of *KRTAP2-3* mRNA expression induced by various stiffness (Fig. S5A–F). Correlation analysis revealed a strong association between actin polymerization and *KRTAP2-3* expression (Fig. 4K). Together, these data suggest that actin polymerization promotes *KRTAP2-3* mRNA expression in response to stiff matrix.

In line with previous data, cytochalasin B treatment promoted ciliary growth and reduced *KRTAP2-3* expression. To further explore the role of *KRTAP2-3*, we examined whether overexpression of *KRTAP2-3* mRNA could counteract the effects of actin depolymerization on ciliogenesis. Surprisingly, when *KRTAP2-3* was exogenously overexpressed, ciliogenesis was inhibited even in the presence of cytochalasin B (Fig. 4L–N), and endogenous *KRTAP2-3* was decreased by treating with cytochalasin B (Fig. S5G). These results indicate that *KRTAP2-3* is a key mediator of actin polarization-dependent regulation of ciliogenesis.

### Actin cytoskeleton tension induces nuclear deformation in response to stiff matrix

**Fig. 5:**
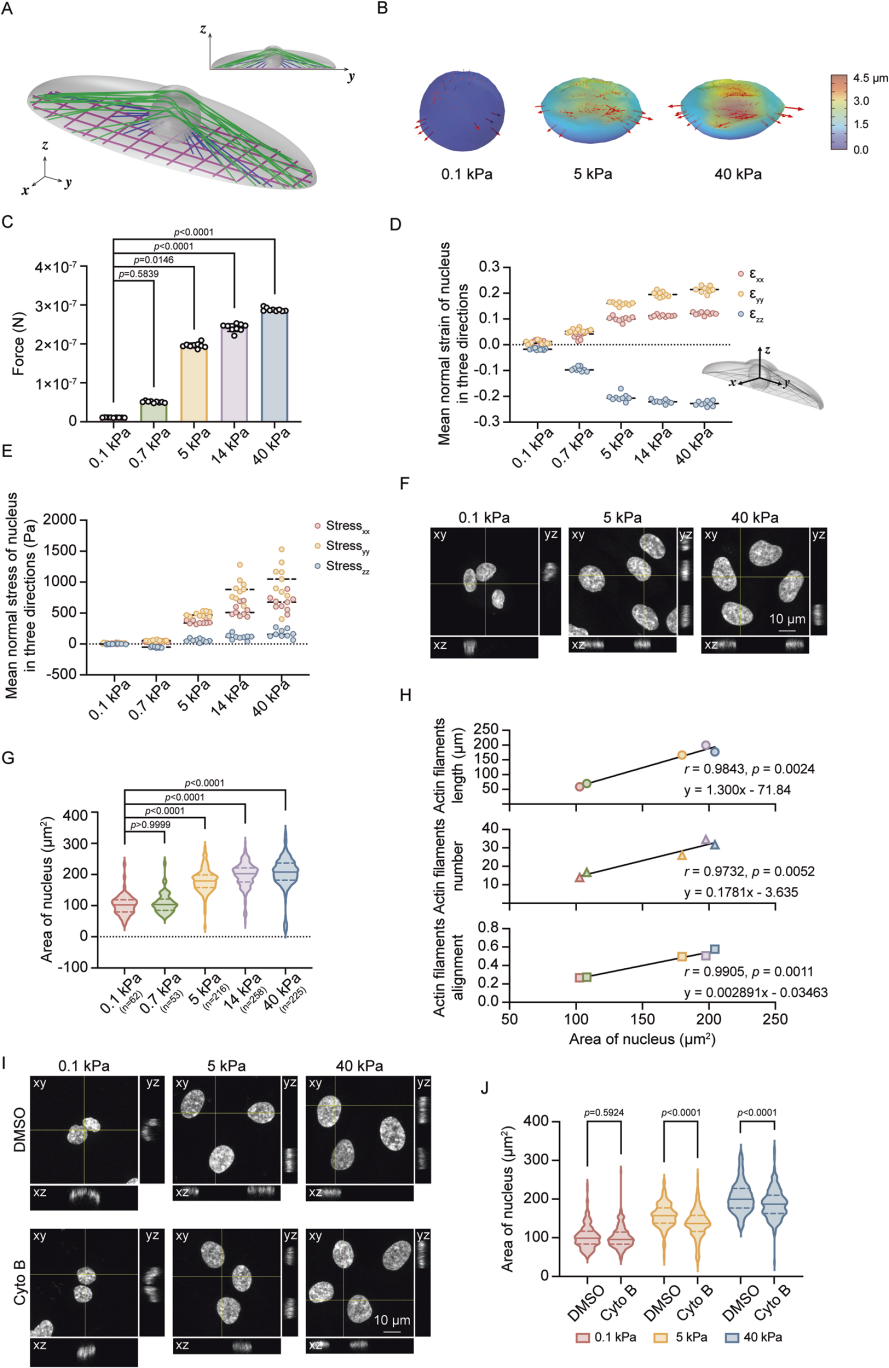
Nuclear deformation regulated by actin cytoskeleton in response to matrix stiffness. **A**–**E** Finite element model shows the relationship between nuclear deformation and force exerted by actin filaments. **A** Model schematic. **B** Force analysis diagram displays the numbers and magnitude of the force by actin filaments. The length of the arrow represents the magnitude of the force. **C** Quantification of total force exerted by actin filaments on the nucleus under varied matrix stiffness. Quantitative data are presented as mean ± SD, Kruskal-Wallis analysis. **D** Mean normal strain in three nuclear directions: *z* (height) and *x*, *y* (nuclear spread area and polarization) in increased matrix stiffness. We defined the major axis of cells as nuclear *y*-axis. **E** Analysis of nuclear normal stress in three directions. We defined the major axis of cells as nuclear *y*-axis. **F**, **G** RPE-1 cells were stained with DAPI (gray) to analyze nuclear area (μm^2^) (*n* = nucleus number). **F** Representative images showing nuclear morphology under varied matrix stiffness (Scale bars, 10 μm), and **G** quantification of nuclear area (μm^2^). Quantitative data are presented as mean ± SD, Kruskal–Wallis test. Experiments were performed with at least three biological replicates. **H** Correlation analysis between actin filaments characteristics (length, number, and alignment) and the nuclear area. Data are presented as simple linear regression and correlation analysis. **I**, **J** RPE-1 cells grown on matrix of the indicated stiffness were treated with DMSO or cytochalasin B (Cyto B) for 6 h and stained with DAPI (gray) to analyze nuclear area (μm^2^). **I** Representative nuclear images after treatment (Scale bars, 10 μm), **J** Quantification of nuclear area (μm^2^). The cell counts of (**J**) from left to right are as follows: 206, 203, 203, 226, 231, 217. Quantitative data are presented as mean ± SD, two-tailed Student’s *t* test. Experiments were performed with at least three biological replicates.

We investigated how actin polymerization promotes *KRTAP2-3* transcription, hypothesizing that actin filaments tension induces nuclear deformation, which influences chromatin accessibility and gene expression [21, 56–58]. Thus, we developed a finite element model to investigate how matrix stiffness regulates the tension force of actin filaments that induces the deformation of nucleus (Fig. 5A, B). The model precisely calculated the increase in the total force exerted by actin filaments on the nucleus as matrix stiffness rises (Fig. 5C). Strain analysis revealed that nuclei flattened more severely along the *z*-axis and spread more broadly in the *x*- and *y*-directions as matrix stiffness increased (Fig. 5D). Importantly, nuclei displayed stronger polarization, with higher strain along the major axis compared to that along the minor axis (Fig. 5D). Nuclear normal stress also has the same increasing trend in three directions coordinated with total force exerted by actin filaments (Fig. 5E). The change of the nuclear area was used as an indicator of nuclear deformation, and our data revealed a positive correlation between nuclear area and actin filaments metrics (length, number, alignment) across varying matrix stiffnesses (Fig. 5F–H). Furthermore, treatment with cytochalasin B decreased nuclear area on stiff matrix (Fig. 5I, J). These data further confirm that nuclear deformation induced by changes in matrix stiffness is a response to force exerted by actin filaments.

### Nuclear deformation induced by stiff matrix enhances *KRTAP2-3* chromatin accessibility

**Fig. 6:**
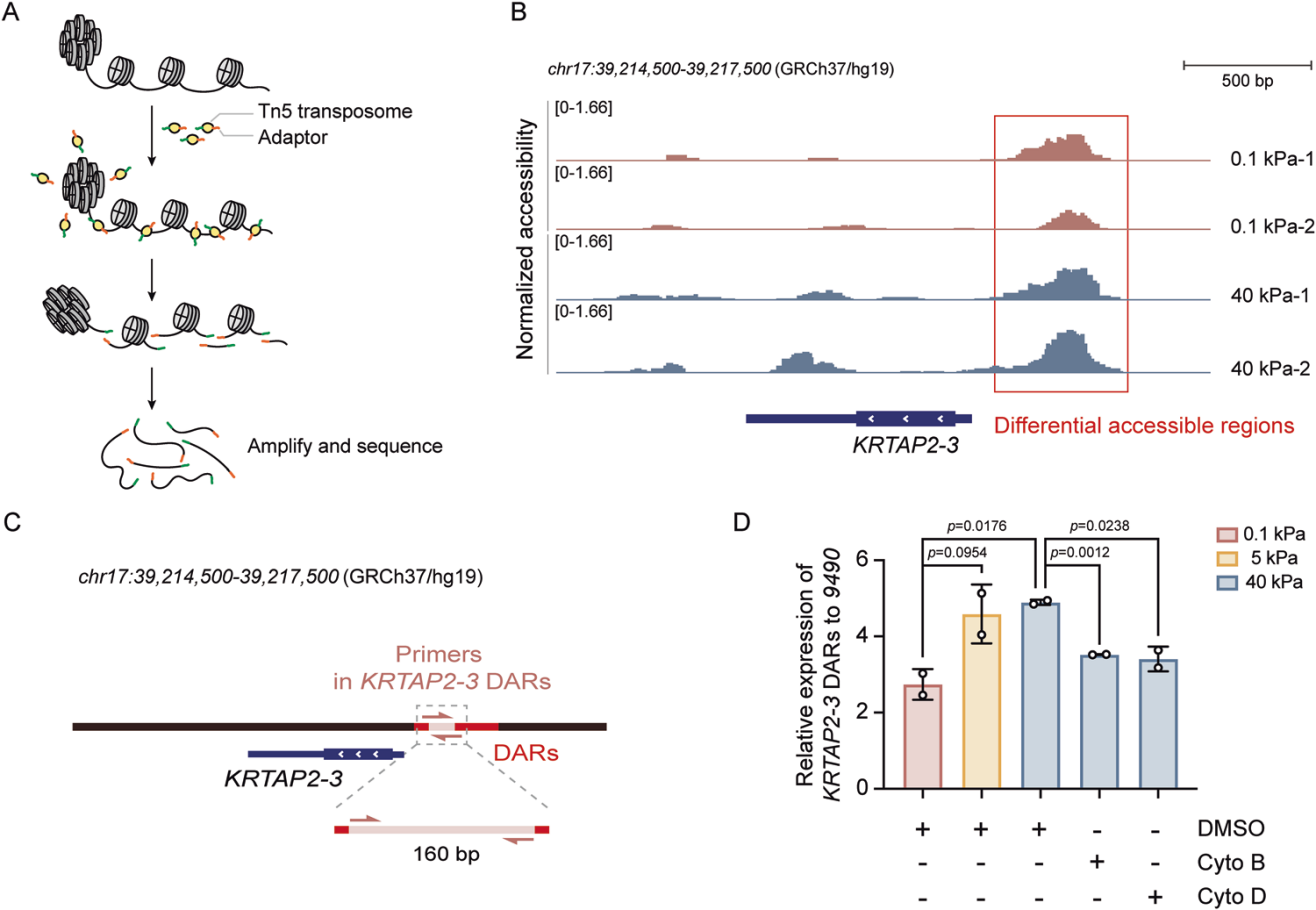
Chromatin accessibility changes in response to matrix stiffness. **A** ATAC-seq workflow schematic. **B** Differential accessible region upstream of the *KRTAP2-3* gene locus between 0.1 and 40 kPa matrix. Scale bars, 500 bp. **C** The locations of primers for ATAC-qPCR are shown as indicated. **D** RPE-1 cells grown on matrix of the indicated stiffness were treated with DMSO, cytochalasin B (Cyto B), or cytochalasin D (Cyto D) for 6 h were analyzed by ATAC-qPCR. Data show allele-specific accessibility at the *KRTAP2-3* locus under different treatments. Normalized to 9490. 9490 region is a constant gene locus (located at chr1: 241683128-241683278 according to hg19) in 0.1/40 kPa matrix stiffness according to ATAC-seq data, which is designed by ATAC Primer Tool (APT). Quantitative data are presented as mean ± SD, two-tailed Student’s *t* test. Experiments were performed with at least two biological replicates.

Given our findings that actin cytoskeleton tension regulates *KRTAP2-3* mRNA levels, we investigated whether this regulation is connected to chromatin remodeling mediated by nuclear deformation. To explore this, we performed ATAC-seq to measure chromatin accessibility by inserting adapters into open genomic regions (Fig. 6A). Comparative analysis of the chromatin accessibility profiles revealed a more accessible region located upstream of the *KRTAP2-3* gene locus in cells on the stiff matrix (40 kPa) compared to the soft matrix (0.1 kPa) (Fig. 6B). These results indicate that chromatin accessibility at the *KRTAP2-3* locus is modulated by matrix stiffness.

To further assess the influence of matrix stiffness and actin polarization on *KRTAP2-3* accessibility, we analyzed chromatin accessibility changes under disrupted actin polarization across varying stiffness levels. Cells were cultured on matrix with stiffnesses of 0.1 kPa, 5 kPa and 40 kPa, and treated with cytochalasin B/D on 40 kPa matrix to disrupt actin polarization. To validate the chromatin accessibility changes observed in ATAC-seq, we carried out ATAC-qPCR with primers designed using the ATAC Primer Tool developed by Howard’s group [59] (Fig. 6C). First, ATAC-qPCR confirmed a significant increase in chromatin accessibility upstream of *KRTAP2-3* in cells cultured on the stiff matrix, consistent with the ATAC-seq results (Fig. 6D). Treatment with cytochalasin B or D led to a marked reduction in accessibility at this region, indicating that the stiffness-induced chromatin opening is dependent on an intact actin cytoskeleton (Fig. 6D). Together, these results establish that matrix stiffness modulates chromatin accessibility at the *KRTAP2-3* locus, likely through actin-mediated nuclear tension and mechanotransduction.

## Discussion

**Fig. 7:**
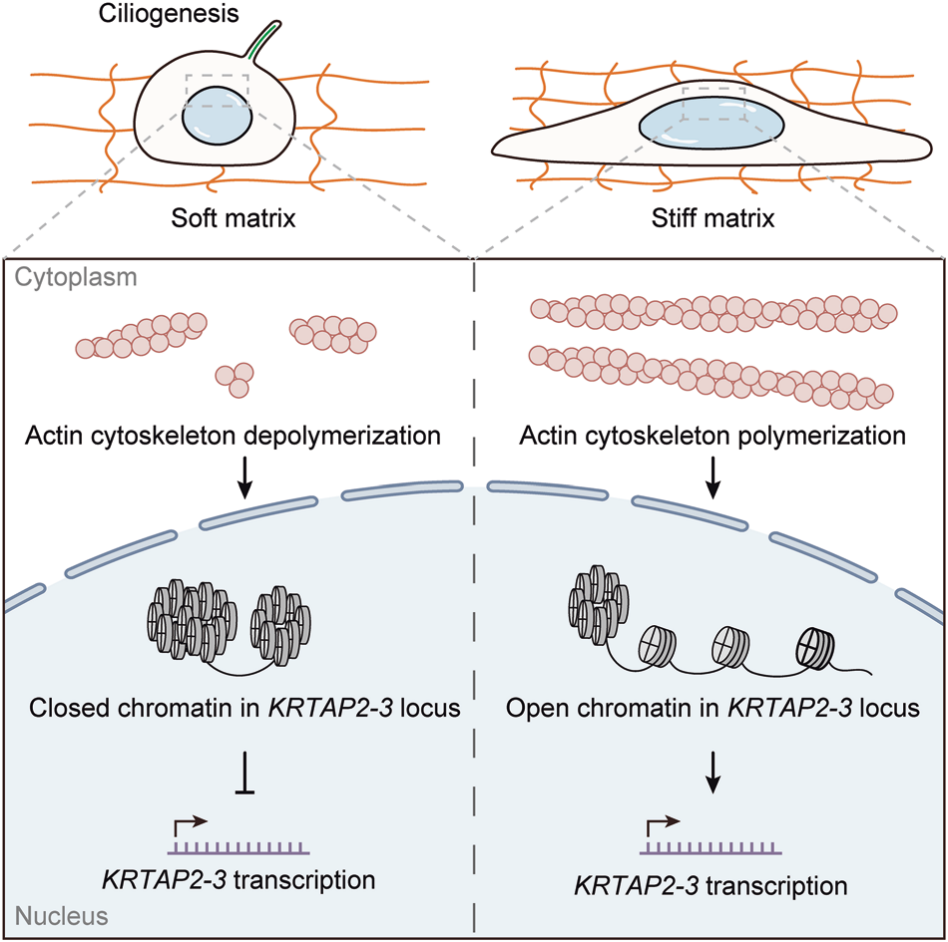
Work model. Stiff matrix triggers cellular actin polymerization, which then induces nuclear deformation. This deformation upregulates chromatin accessibility at the *KRTAP2-3* gene locus, enhancing its transcription and subsequently inhibiting ciliogenesis.

This study elucidates a biomechanical pathway by which matrix stiffness regulates primary cilia formation through actin polarization and nuclear deformation. Our data show that stiff matrix inhibits ciliogenesis by promoting actin polarization and upregulating *KRTAP2-3* mRNA levels. We show that the increased tension in the actin cytoskeleton induces greater nuclear deformation, which enhances chromatin accessibility at the *KRTAP2-3* gene locus, leading to its upregulation and subsequent transcription. Thus, we reveal that the actin cytoskeleton serves as a key mediator of mechanotransduction, linking matrix stiffness, nuclear deformation, and ciliogenesis (Fig. 7).

Changes in ECM stiffness are central to tissue development, homeostasis, and pathological remodeling. During development and organogenesis, ECM mechanics guide cell fate decisions and coordinated tissue behavior [60, 61], whereas pathological ECM stiffening is a hallmark of fibrosis and cancer [62]. Although ECM stiffness is known to regulate a wide range of cellular behaviors [63], how mechanical cues influence primary cilia—key organelles for sensing and transducing developmental and homeostatic signals—has remained poorly understood. Our findings place primary ciliogenesis within the framework of tissue mechanobiology by identifying matrix stiffness as a direct upstream regulator. While previous studies reported stiffness-dependent changes in cilia length, the underlying mechanisms were unclear [64]. Here, we demonstrate that matrix stiffening suppresses ciliogenesis through an actin tension–driven pathway that couples cytoskeletal polarization to nuclear deformation and transcriptional regulation. This work provides a conceptual link between ECM stiffening and cilia loss, suggesting how mechanical changes in developing or diseased tissues may reshape cilia-dependent signaling and cellular function.

KRTAP2-3, a member of the KRTAP2 family, has previously been implicated in mediating TGF-β‘s effects on cancer cell motility and proliferation [65]. However, its role in other cellular processes, including ciliogenesis, has not been fully explored. We identify *KRTAP2-3* as a mechanosensitive gene, upregulated by matrix stiffness, whose increased expression inhibits ciliogenesis. Notably, *KRTAP2-3* mRNA levels correlate inversely with the extent of ciliation, making it a previously uncharacterized negative regulator of ciliogenesis and linking mechanical cues to gene regulation.

Our study also establishes a novel connection between nuclear deformation, chromatin remodeling, and ciliogenesis. Actin polarization plays a pivotal role in nuclear deformation, which alters chromatin accessibility and impacts gene expression. By integrating confocal imaging with finite element modeling, our study demonstrates that actin cytoskeleton tension induces nuclear architectural changes, enhancing chromatin accessibility at the *KRTAP2-3* locus and promoting its transcription. The observed correlation between actin polarization and *KRTAP2-3* expression reveals the gene’s sensitivity to mechanical cues, thereby establishing a direct link between cytoskeletal polarization and gene regulation. These findings suggest that matrix stiffness influences the ciliary phenotype through an actin tension-driven mechanism involving nuclear deformation and chromatin remodeling, revealing a previously unrecognized layer of mechanotransduction during ciliogenesis.

Despite these findings, several limitations warrant further investigation. First, while our study identifies *KRTAP2-3* as a novel inhibitor of ciliogenesis, the precise mechanism by which *KRTAP2-3* exerts its effects on ciliogenesis remains unclear. Second, the mechanism by which actin tension-induced chromatin remodeling triggers *KRTAP2-3* transcription is still unknown—perhaps through the recruitment of specific transcription factors. Future research aimed at identifying these transcription factors would significantly advance our understanding of this process. Additionally, matrix stiffness is implicated in various pathological conditions, including fibrosis and cancer. Given our finding that stiffness regulates *KRTAP2-3* expression and ciliogenesis, and considering the critical role of cilia in numerous signaling pathways, it is important to explore whether these processes could be targeted therapeutically in these diseases.

## Supplementary information

reproducibility checklist

Supplementary Figures

Supplementary Table 1: List of differentially expressed genes.

Supplementary Table 2: List of primers used in this study.

Supplementary Table 3: List of simulation parameters.

## Data Availability

RNA-seq data of RPE-1 cells cultured on different matrix stiffness and ATAC-seq data of cells cultured on 0.1 or 40 kPa matrix reported in this paper have been deposited in the Genome Sequence Archive (Genomics, Proteomics and Bioinformatics 2025) in National Genomics Data Center (Nucleic Acids Res 2025), China National Center for Bioinformation / Beijing Institute of Genomics, Chinese Academy of Sciences (GSA-Human: HRA009360) that are publicly accessible at https://ngdc.cncb.ac.cn/gsa-human [66, 67]. Any additional information required to reanalyze the data reported in this paper is available from the lead contact upon request.
