# Supplementary material for: Stiff matrix-induced KRTAP2-3 expression suppresses ciliogenesis via actin tension-driven chromatin remodeling
Source: Cell Death Dis. 2026 Apr 2;17(1):443. doi: 10.1038/s41419-026-08678-1 (PMC13168523; doi:10.1038/s41419-026-08678-1)
Supplement: Supplementary file 2 — Supplementary Figures [file 41419_2026_8678_MOESM2_ESM.docx]

Document S1. Figures S1–S6


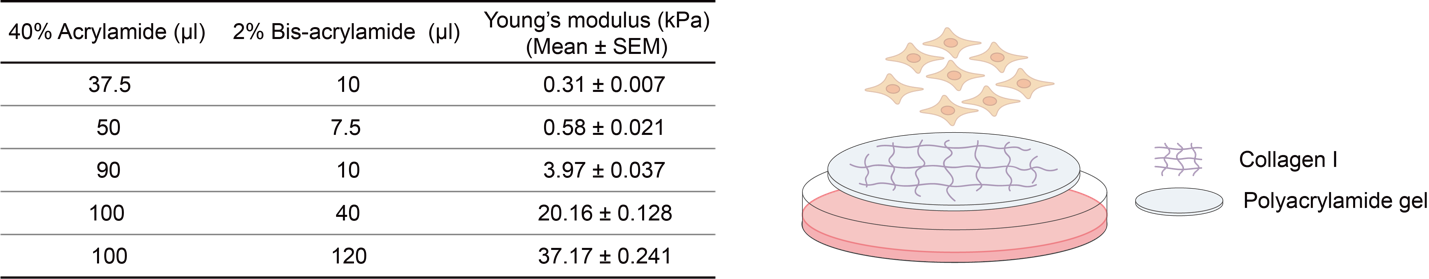


**Figure S1.** **Polyacrylamide gel cell culture model**

Cells were seeded on top of polyacrylamide (PA) gels, pre-coated with collagen to promote cell adhesion before experimentation.


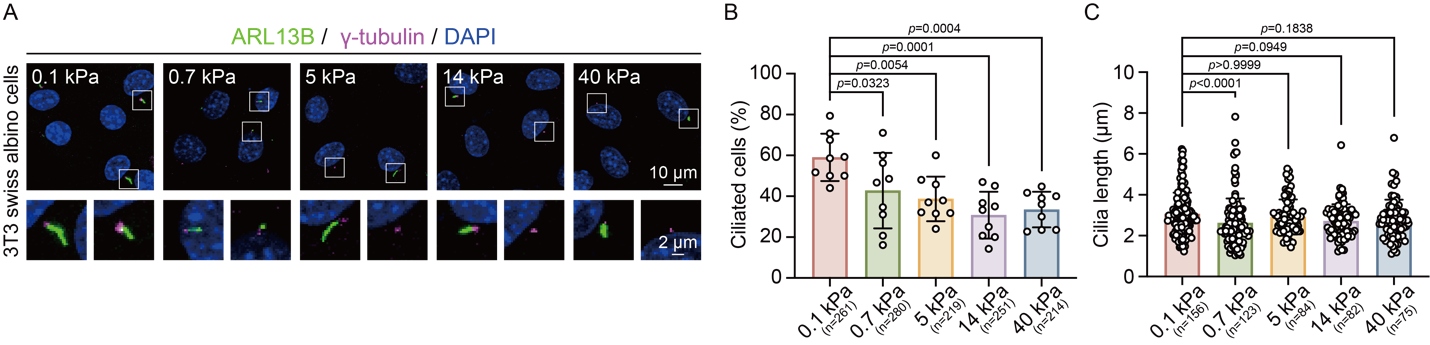


**Figure S2. Matrix stiffness suppresses primary cilia formation.**

(A-C) 3T3 cells were cultured on matrix of increasing stiffness for 12 hours and immunostained to analyze cilia formation. (A) Representative images show cells stained with anti-ARL13B (green) for cilia and anti-γ-tubulin (magenta) for basal bodies. Scale bars, 10 μm (upper panels) and 2 μm (lower panels). (B) Quantification of the percentage of ciliated cells under varied matrix stiffness (n = cell number). Quantitative data are presented as mean±SD, ordinary ANOVA analysis. (C) Measurement of cilia length (n = cilia number). Quantitative data are presented as mean±SD, Kruskal-Wallis analysis.


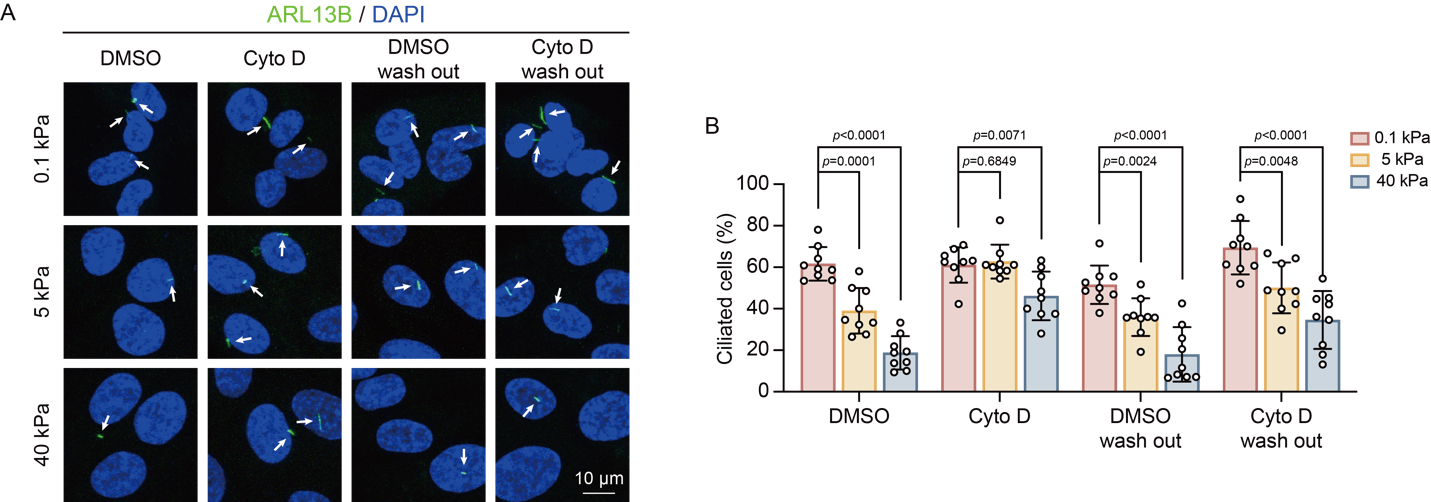


**Figure S3. Stiff matrix inhibits ciliogenesis through actin polymerization.**

(A and B) RPE-1 cells grown on matrix of the indicated stiffness were treated with DMSO or cytochalasin D (Cyto D) for 6 hours, followed by a 6-hour recovery, and stained for cilia. (A) Representative images of cilia stained with anti-ARL13B (green). Scale bars, 10 μm. (B) Quantification of the percentage of ciliated cells post-treatment. Quantitative data is presented as mean±SD, two-tailed Student’s t-test.


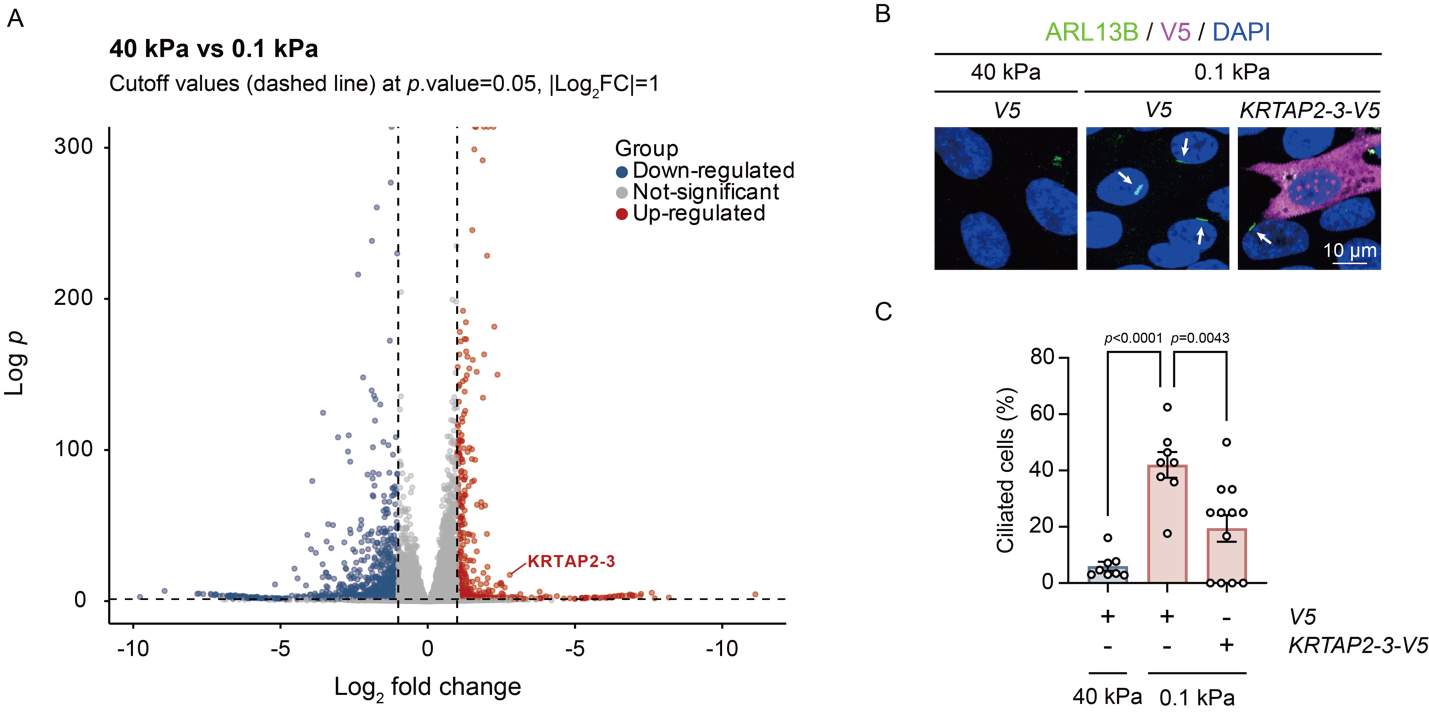


**Figure S4. Overexpression of KRTAP2-3 inhibits ciliogenesis on a soft matrix.**

(A) Volcano plot of differentially expressed genes in cells cultured on 40 kPa versus 0.1 kPa matrix. The plot displays Log₂ fold change versus Log₁₀ (*p* value) for all detected genes. Dashed lines indicate the thresholds for differential expression (|Log₂ fold change| = 1 and *p* = 0.05).

(B and C) RPE-1 cells were transfected with *KRTAP2-3-V5* mRNA for 12 h and cultured on matrix of the indicated stiffness under standard culture conditions, followed by immunofluorescence analysis. (B) Representative images showing cells stained with anti-ARL13B (green) to label primary cilia and anti-V5 (red) to detect KRTAP2-3-V5. Scale bars, 10 μm. (C) Quantification of the percentage of ciliated cells. Data are presented as mean ± SD; statistical significance was assessed using a two-tailed Student’s t-test.


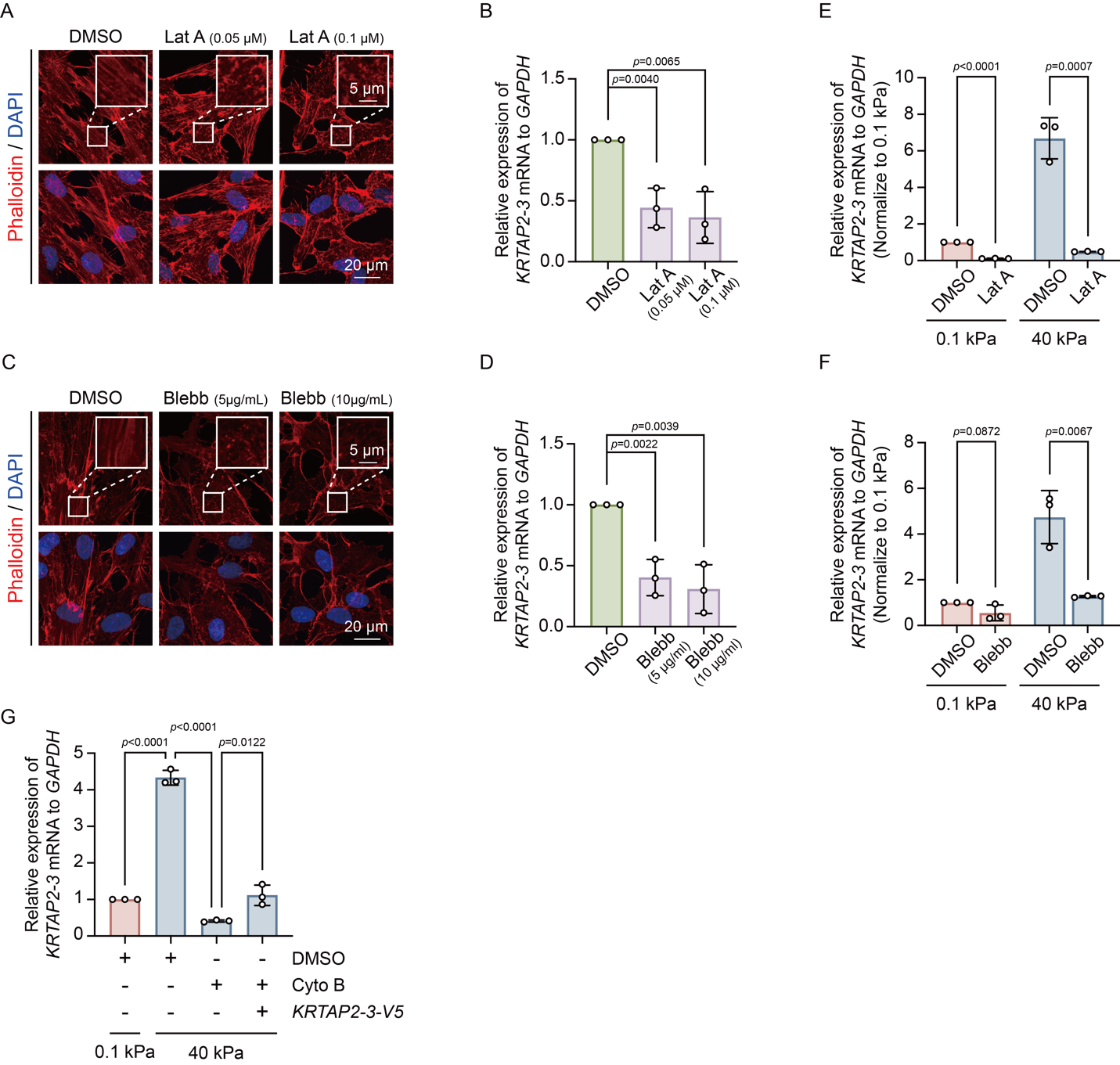


**Figure S5. Regulation of ciliogenesis by KRTAP2-3 in response to actin cytoskeleton changes.**

(A-D) RPE-1 cells were treated with DMSO, latrunculin A (Lat A), or blebbistatin (Blebb) for 6 hours and then analyzed by immunofluorescence and qRT-PCR. (A and C) Representative actin images after treatment. Scale bars, 5μm (upper panels) and 20 μm (lower panels). (B and D) qRT-PCR analysis of *KRTAP2-3* expression, normalized to *GAPDH*.

(E and F) RPE-1 cells cultured on the indicated matrix of 0.1 and 40 kPa stiffness were treated with DMSO, Lat A, or Blebb for 6 hours, followed by qRT-PCR of *KRTAP2-3* expression (*GAPDH* as control).

(G) RPE-1 cells transfected with *KRTAP2-3-V5* mRNA were cultured on matrix of the indicated stiffness and treated with either DMSO or cytochalasin B (Cyto B) for 12 hours. qRT-PCR analysis was conducted to measure endogenous *KRTAP2-3* expression, with data normalized to *GAPDH*. Quantitative data is presented as mean±SD, two-tailed Student’s t-test.


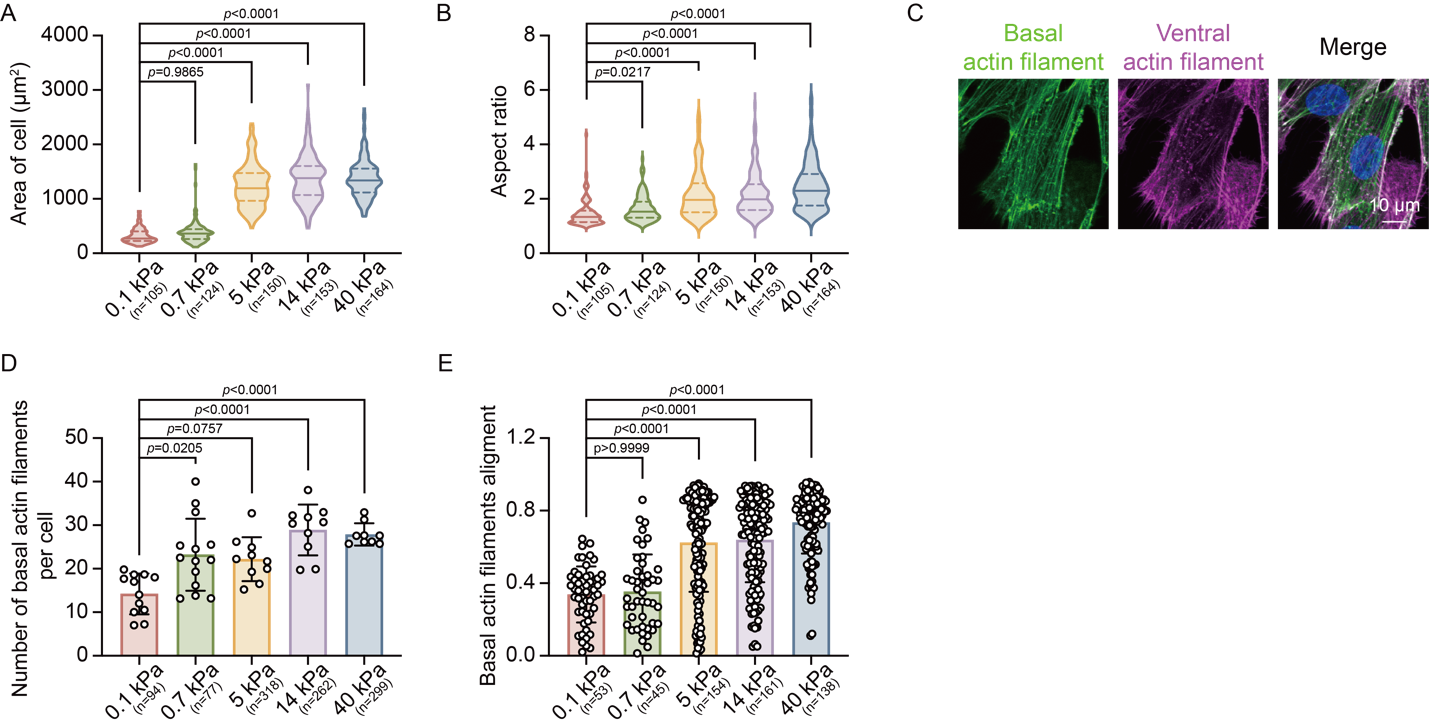


**Figure S6. Measurement of simulation parameters in the finite element model.**

(A and B) Quantification of cell area (A) and aspect ratio (B) under varied matrix stiffness. Quantitative data are presented as mean ± SD, Kruskal-Wallis analysis.

(C) Image shows z-direction projection images of actin filaments at different heights. Basal actin filaments are in green. Ventral actin filaments are in magenta. Nucleus is in blue. Scale bars, 10 μm.

(D and E) RPE-1 cells were cultured on matrix of increasing stiffness for 12 hours and stained with phalloidin to analyze basal actin filaments. (D) Quantification of basal actin filament number, and (E) quantification of basal actin filament alignment. Quantitative data are presented as mean ± SD, Kruskal-Wallis analysis
